# Editorial: Extracellular Vesicle-Mediated Processes in Cardiovascular Diseases

**DOI:** 10.3389/fcvm.2018.00133

**Published:** 2018-09-19

**Authors:** Rory R. Koenen, Elena Aikawa

**Affiliations:** ^1^Department of Biochemistry, Cardiovascular Research Institute Maastricht, Maastricht University, Maastricht, Netherlands; ^2^Cardiovascular Division, Department of Medicine, Center for Excellence in Vascular Biology, Harvard Medical School, Brigham and Women's Hospital, Boston, MA, United States

**Keywords:** microparticle, atherosclerosis, vascular calcification, diagnostic, biomarker, heart valve

The review articles provided in this research topic highlight extracellular vesicles (EV) and the processes that they regulate in cardiovascular diseases. Even if landmark achievements have initially been within the field of cancer biology, the importance of EVs in cardiovascular disease is impossible to overlook. To provide the readers with a state-of-the-art knowledge in the field of EV-mediated processes in cardiovascular diseases, we have composed a broad collection of contributions from experts in the field. In this research topic we cover several aspects of EVs, from their (patho-)physiologic functions in cardiac development and regeneration, their role in angiogenesis and atherosclerosis, their involvement in diabetes-related cardiovascular complications, to the exciting implications in cardiovascular calcification. In addition, the optimization of EV measurement methods and their use as biomarkers and potential therapeutics have been highlighted.

Having been disregarded as mere cellular debris for decades, EVs are increasingly being appreciated as integral mediators of cell-to-cell communication. EVs can be classified into three major groups differentiated by size and origin: exosomes (40–100 nm), consisting of cytoplasmic compartments released by exocytotic processes; microvesicles (100–500 nm), generated by budding of the plasma membrane; and apoptotic bodies (~1,000 nm), that are shed by cells undergoing programmed death. As every cell is able to release EVs, their body-wide physiologic and pathologic relevance is self-evident. The proteins and genetic material of EVs may reflect the conditions of the parental cells. To carry message to distal parts of the body, loaded with concentrated cargo miniscule EVs that able to move easily in extracellular space and circulation are likely intendent for a specific function such as interaction via carrying diverse molecular payload (e.g., nucleic acids, bioactive lipids, and proteins) to distal or neighboring recipient cells. This essential biological function of EVs helps to maintain tissue homeostasis in health or contribute to the disease when vesicles acquire pathological properties.

In the contribution by Gross and Zelarayán, the roles of Wnt signaling in cardiac development and during cardiac stress are discussed. The transmission of Wnt signals between cells has been shown to occur through EV. Given the importance of Wnt signaling for general physiology, this likewise highlights EV's physiological relevance, being integral signaling transporters.

Changes in the EV concentrations during cardiovascular diseases have been observed in many studies. Since platelets are among the most abundant blood cells, they represent a major source of circulating EVs. In the article by Zaldivia et al., the functions of EVs derived from platelets are discussed in the pathogenesis of venous thrombosis, atherosclerosis, and myocardial infarction. Yet

EVs from various cellular origins are also recognized as mediators of cellular crosstalk, particularly in atherosclerosis and angiogenesis. This is discussed in the comprehensive reviews by Badimon et al. and van der Vorst et al., together with the possible exploitation of EVs as prognostic or diagnostic biomarkers, or as therapeutics. The overview by Gustafson et al. expands the discussion by focusing on diabetic complications such as cardiomyopathy and atherosclerosis.

A novel role for EVs was identified in the process of cardiovascular calcification, a complication of atherosclerosis, diabetes, chronic kidney disease, and aortic valve stenosis. Calcifying EVs serve as fundamental building blocks of calcification. In vasculature, calcifying EVs aggregate to form microcalcifications contributing to atherosclerosis plaque rupture and subsequent myocardial infarction. In cardiac valves, calcific aggregates result in increased leaflet stiffness causing aortic stenosis, heart failure, and death. As highlighted in the contribution by Bakhshian Nik et al., EVs derived from smooth muscle cells, valvular interstitial cells and macrophages can modulate cardiovascular calcification. Emerging evidence suggests that platelet-derived EVs may also contribute to vascular calcification, ultimately leading to atherothrombotic complications, as outlined by the article from Schurgers et al..

As defined above, exosomes are a subclass of EVs originating from late endosomal compartments. Although initially thought as a mechanism of cellular waste disposal, exosomes are currently well-established mediators of cell-to-cell communication. The exosomes specialized in communication are termed “signalosomes” in the elegant review by Willis et al., harboring molecular cargo capable of influencing the behavior of recipient cells. Such signalosomes act as immunomodulators in models of lung disease, raising an intriguing possibility for use of purified signalosome-type exosomes for a clinical therapeutic application. The hurdles and perspectives for EV therapy are extensively outlined in this review.

Among such hurdles are the proper purification, potency determination and quantification of EVs, which is largely due to their small size. A novel protocol for the size and count measurement of EV between 50 and 120 nm is presented by Parsons et al.. This promising methodology will improve the accuracy of EV characterization in various biologic fluids from both healthy and diseased individuals.

Accurate categorization of EVs is also crucial for their meaningful implementation as biomarkers. The review by Dickhout and Koenen provides an overview of the prerequisites of the use of EVs as biomarkers, dealing with methods and issues of isolation, determination, and classification. An overview of (pre-)clinical studies using EVs as biomarkers is also provided and the potential of EVs as biomarkers for cardiovascular disease is critically discussed.

With the above reviews, we aim to convince the reader that EVs are integral components of an intricate cellular communication system in health and disease, while simultaneously providing a differentiated overview of the current challenges and obstacles, regarding EV characterization and their implementation as biomarkers or therapeutics. Nevertheless, exciting times are ahead of us as EVs are heading straight to the heart of cardiovascular research.

## Author contributions

All authors listed have made a substantial, direct and intellectual contribution to the work, and approved it for publication.

### Conflict of interest statement

The authors declare that the research was conducted in the absence of any commercial or financial relationships that could be construed as a potential conflict of interest.

